# Supplementary material for: Ancestral reconstruction of the MotA stator subunit reveals that conserved residues far from the pore are required to drive flagellar motility
Source: Microlife. 2023 Apr 3;4:uqad011. doi: 10.1093/femsml/uqad011 (PMC10117855; doi:10.1093/femsml/uqad011)
Supplement: uqad011_Supplemental_Files [file uqad011_supplemental_files.zip › MotA_SI_MicroLifeRevisions_20230201.pdf]

Supplementary Material

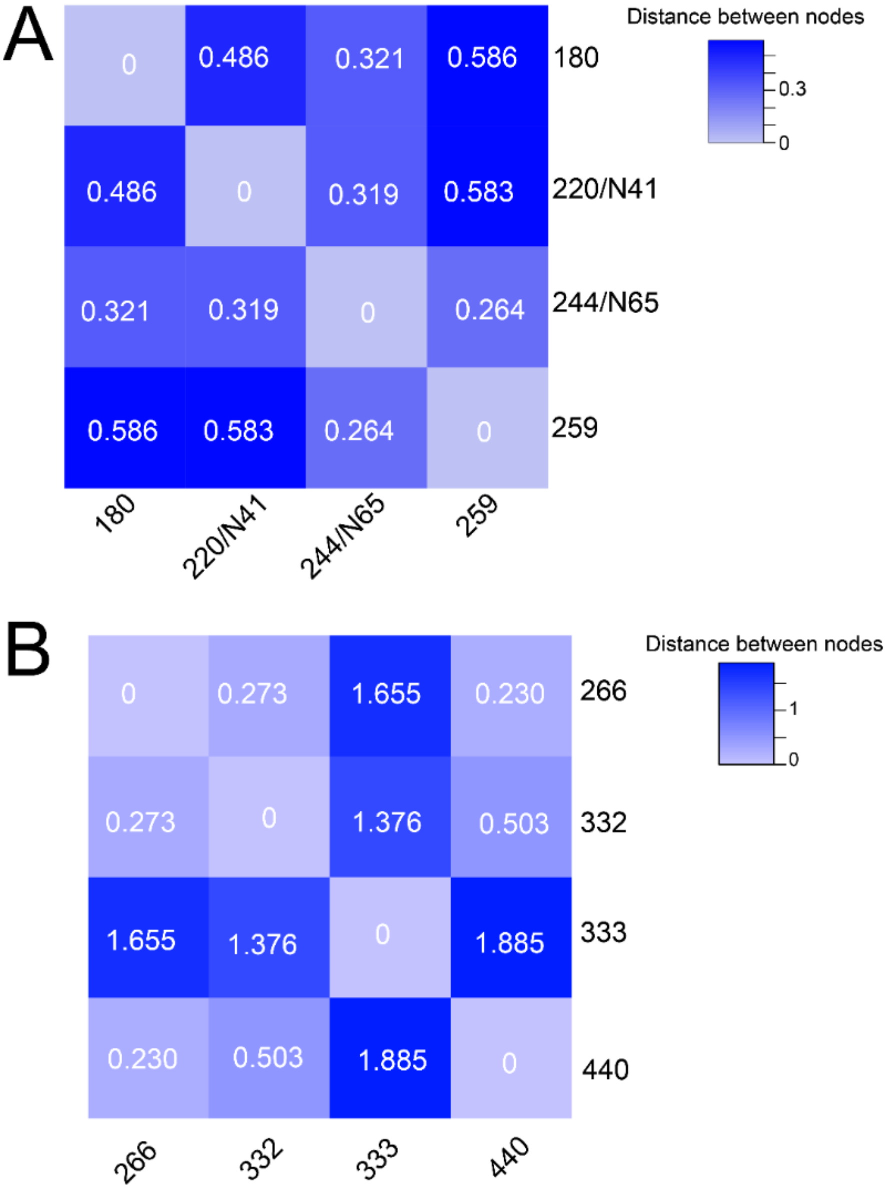

Supplementary Figure 1: Pairwise distances between all selected nodes. (A) Pairwise distances of the nodes (180, 220/N41, 244/N65, and 259) were calculated for the phylogenetic tree of 178 MotA homologs (main Fig. 1A) from the total branch distance of the shortest connection between two nodes. (B) Pairwise distances of the nodes (266, 332, 333, and 440) were likewise calculated from the branch lengths of the phylogenetic tree of 264 MotA homologs (main Fig. 1B). Branch length unit was substitutions per site. The heatmaps were generated in the heatmap2 program from the Galaxy Australia website (https://usegalaxy.org.au/).

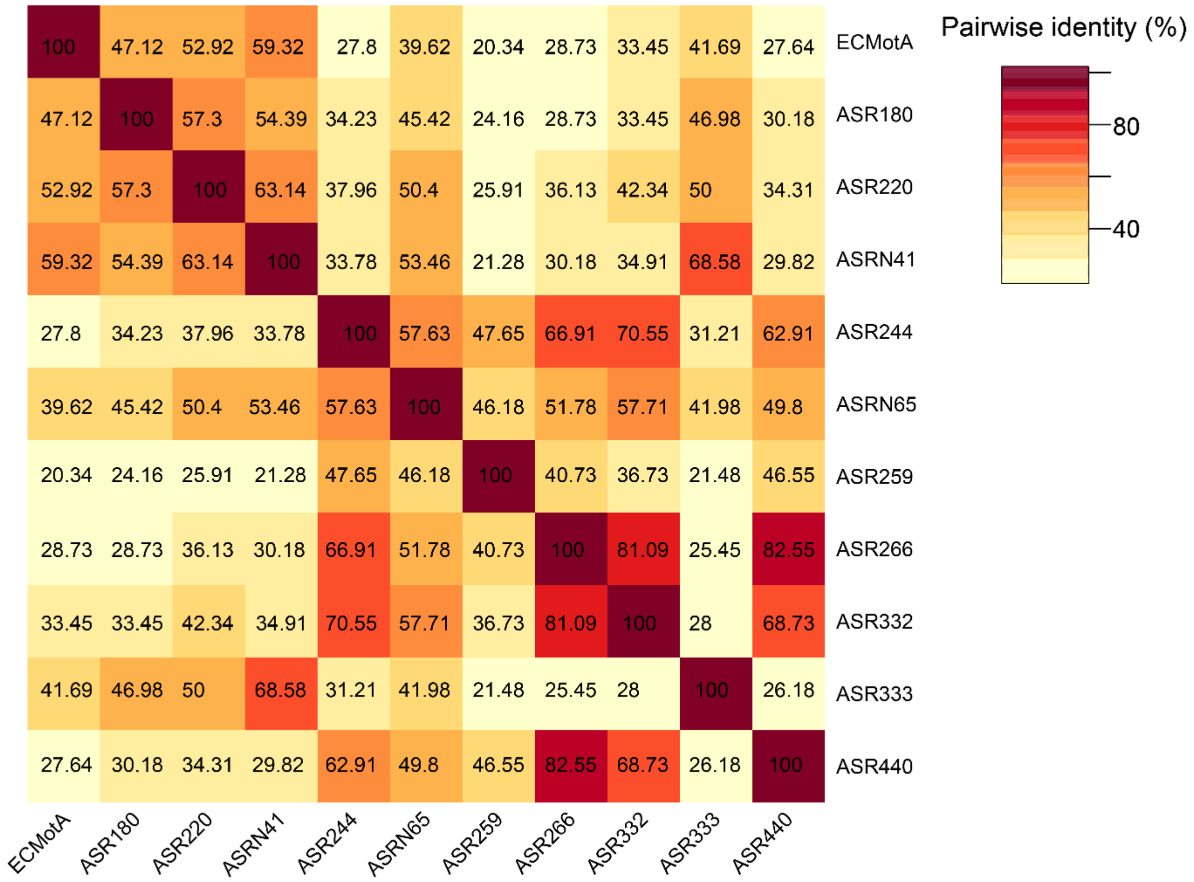

Supplementary Figure 2: Pairwise percentage similarity (identity) displayed as matrix of all ten selected ASRs alongside wild-type *E. coli* MotA. The identity matrix was calculated using Clustal Omega (https://www.ebi.ac.uk/Tools/msa/clustalo/) and the heatmap was generated in the heatmap2 program from the Galaxy Australia website (https://usegalaxy.org.au/).

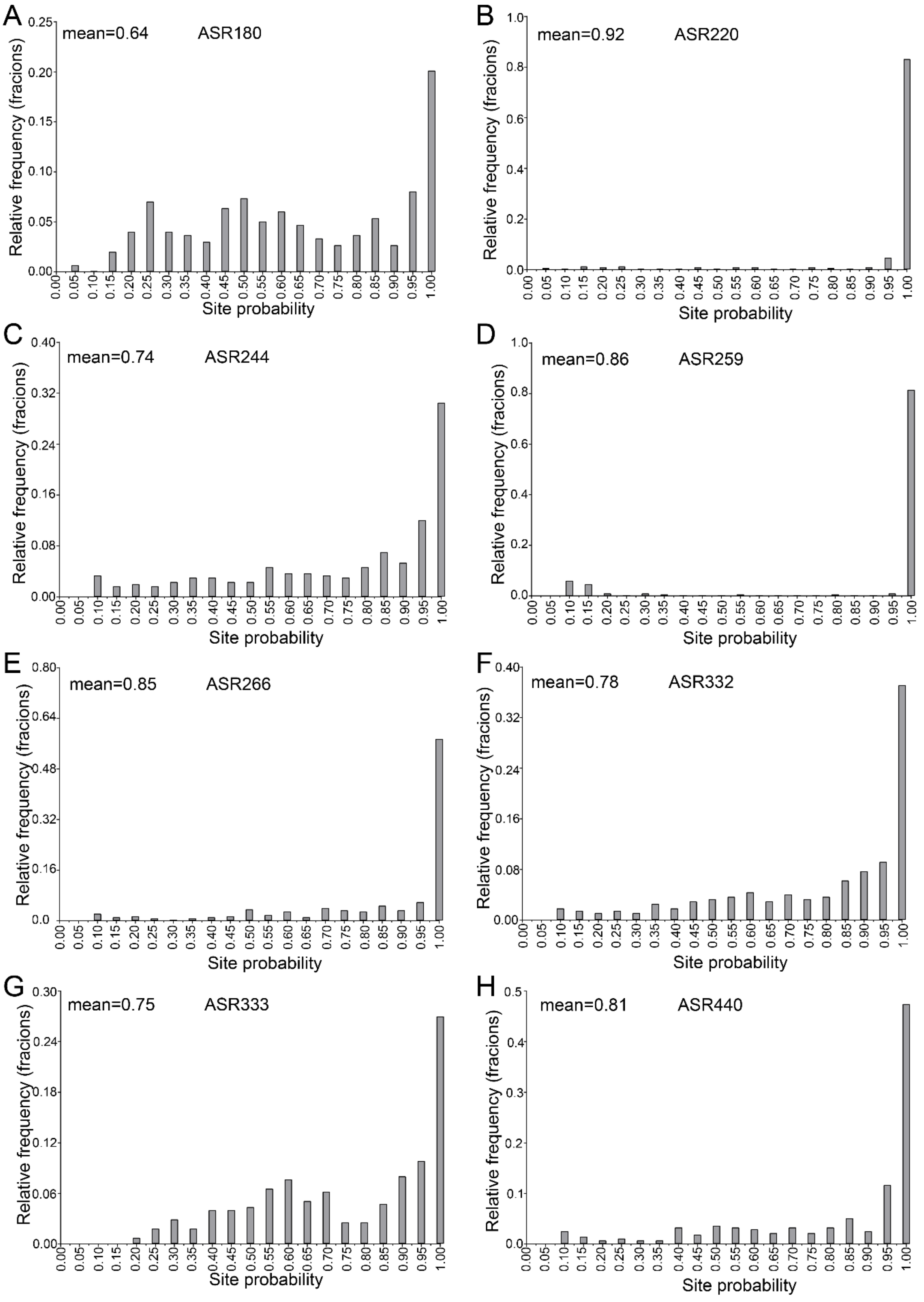

Supplementary Figure 3: Histogram of posterior probabilities from the PAML ancestral reconstruction for most common amino acid at each site, for each ASR, at the 8 selected nodes. Mean probability across all sites is shown inset in top left.

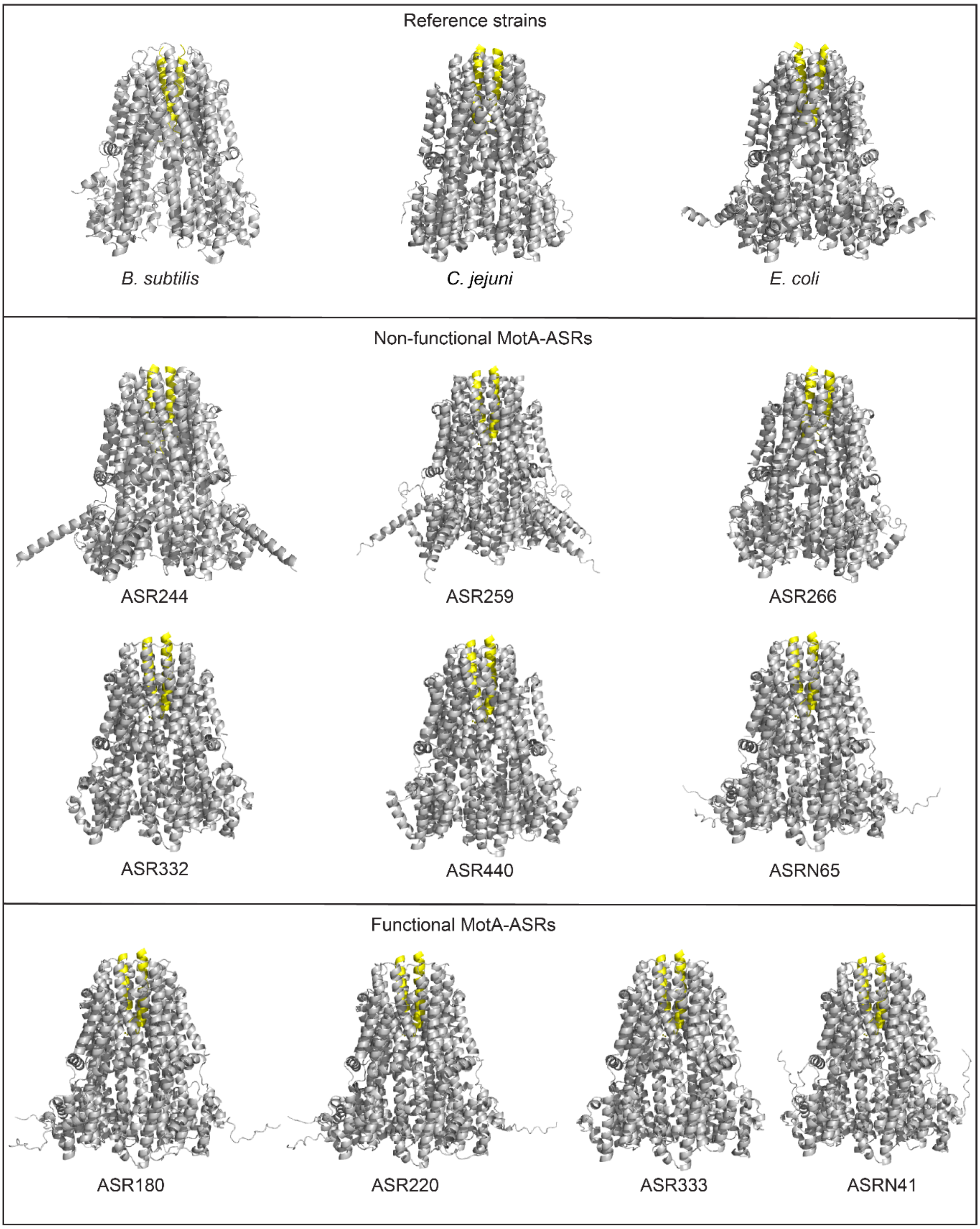

Supplementary Figure 4: ColabFold structural models for MotA-ASRs. Monomer subunits for *E. coli* MotA and each of the ten MotA-ASRs were aligned with pentamer MotA from *C. jejuni* $MotA_5MotB_2$ stator complex structure: PDB-6YKP (Santiveri et al., 2020). MotA pentamers and MotB dimers are show in grey and yellow, respectively. *B. subtilis* structure: PDB-6YSL (Deme et al., 2020), *E. coli* structure from Alphafold model in this work.

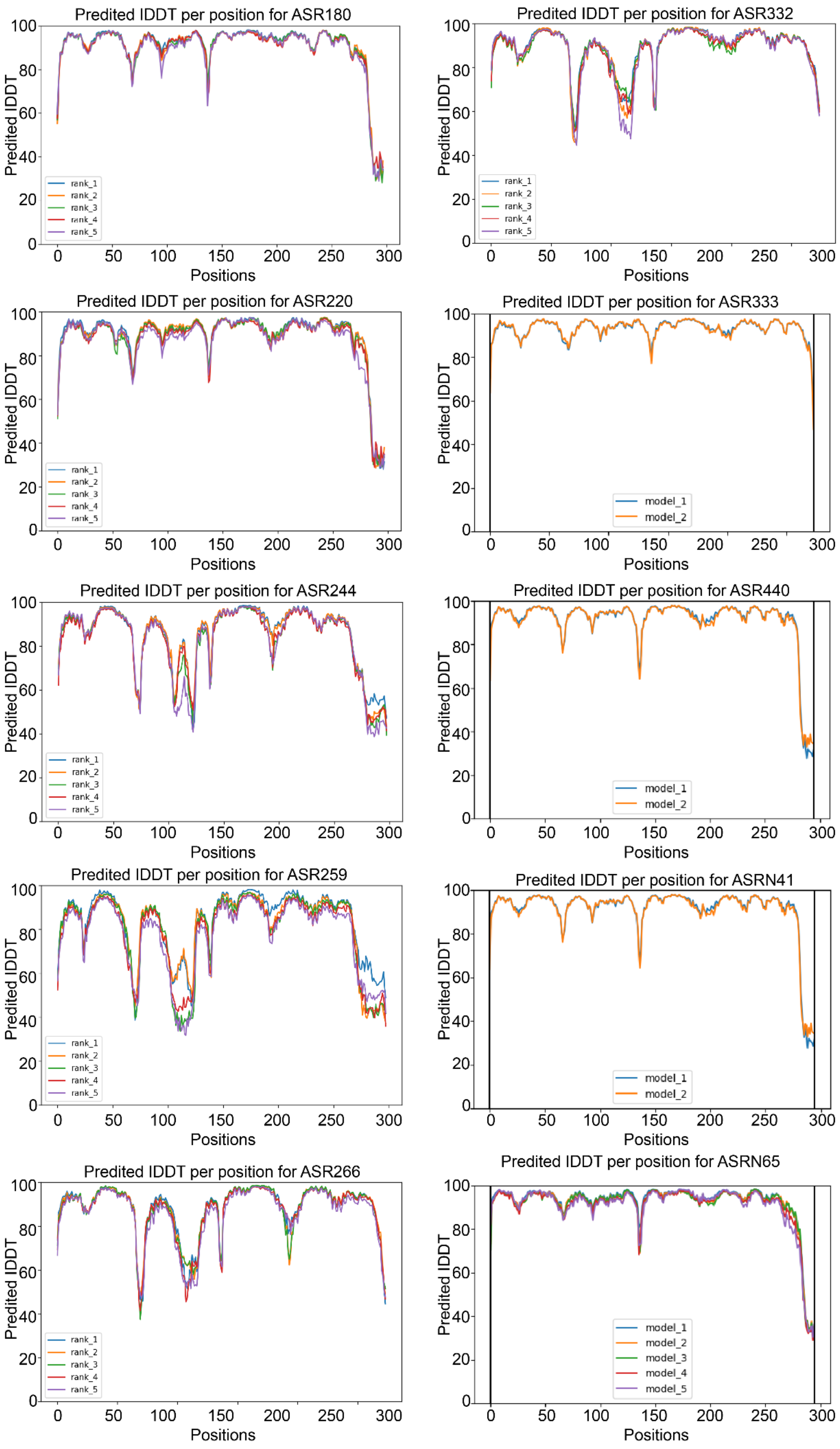
Predited IDDT per position for ASR180
Predited IDDT per position for ASR332
Predited IDDT per position for ASR220
Predited IDDT per position for ASR333
Predited IDDT per position for ASR244
Predited IDDT per position for ASR440
Predited IDDT per position for ASR259
Predited IDDT per position for ASRN41
Predited IDDT per position for ASR266
Predited IDDT per position for ASRN65
Predited IDDT
Positions
rank_1
rank_2
rank_3
rank_4
rank_5
model_1
model_2
model_3
model_4
model_5

Supplementary Figure 5: Predicted LDDT per position for the Colabfold models of MotA-ASRs. All the plots of the MotA-ASRs show the predicted LDDT per position for the 2-5 models obtained from Colabfold output. The top-ranked models were selected for each MotA-ASR and aligned with the *C. jejuni* MotAB complex (PDB:6YKP) shown in Supplementary Figure 4.

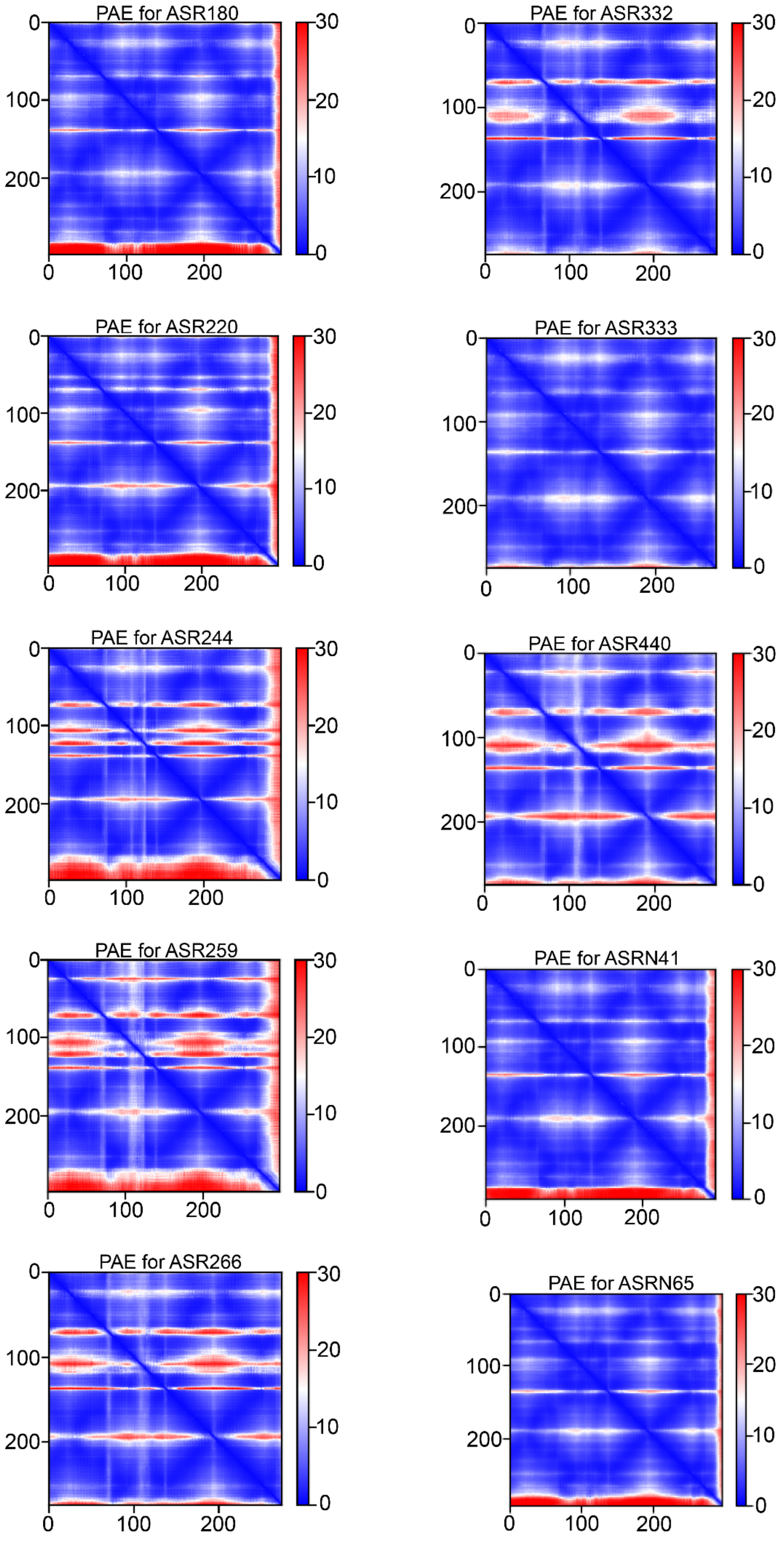
PAE for ASR180
PAE for ASR332
PAE for ASR220
PAE for ASR333
PAE for ASR244
PAE for ASR440
PAE for ASR259
PAE for ASRN41
PAE for ASR266
PAE for ASRN65

Supplementary Figure 6: Predicted aligned errors (PAE) for the MotA-ASR Colabfold models showed in Supplementary Figure 4.

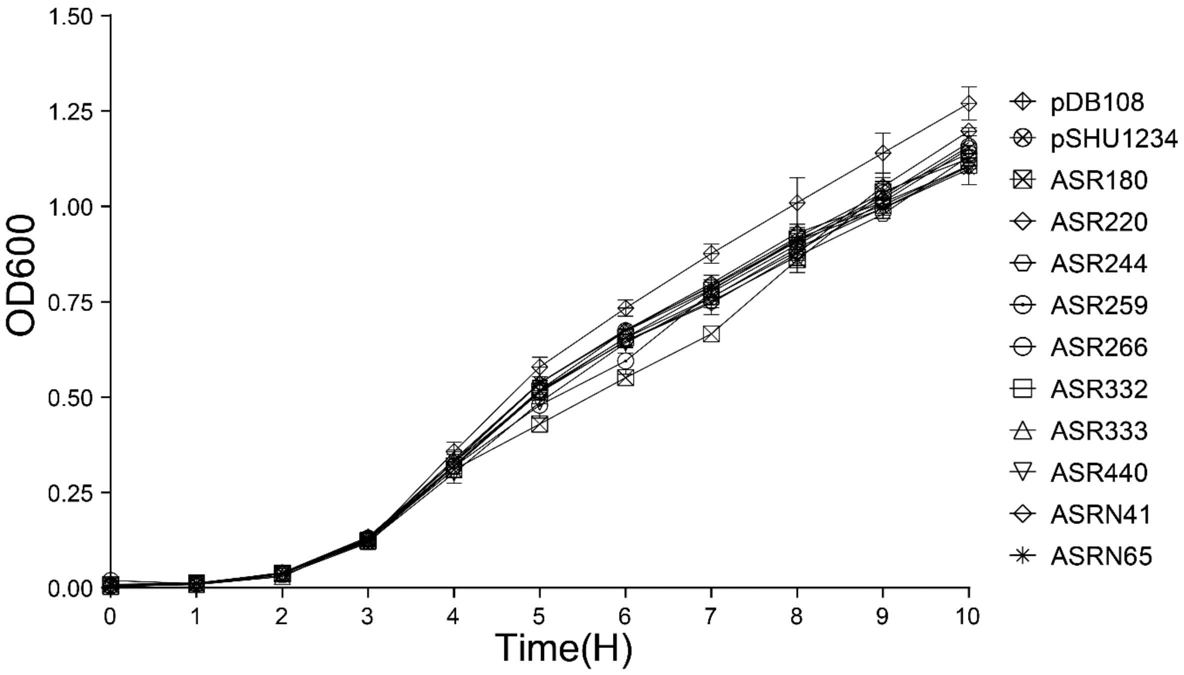

Supplementary Figure 7: Growth curve of MotA-ASRs in the presence of WT *E. coli* MotB. Ten MotA-ASRs transformed into a Δ*motA* RP437 strain and the controls, pSHU1234 (*pomApotB*) and pDB108 (*motAmotB*) transformed into a Δ*motAmotB* RP437. All of the transformed cells were grown in LB broth with appropriate antibiotic selection and 0.2% arabinose in a 96-well microtiter plate, and the OD was measured hourly.

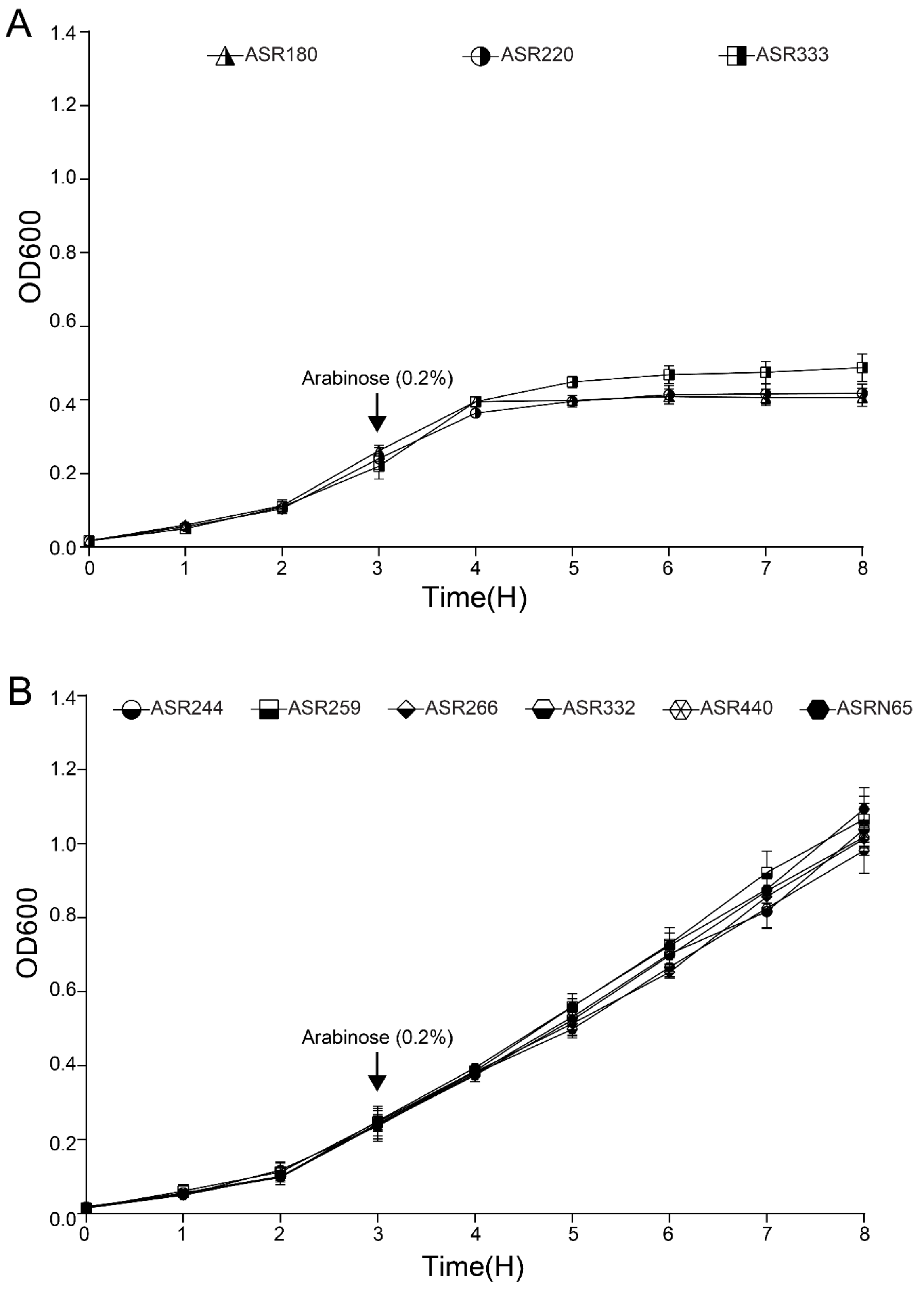

Supplementary Figure 8: Growth curve of MotA-ASRs in the presence of plug deleted (Δ51-70) WT *E. coli* MotB. (A) and (B) are showing the growth curve of the functional MotA-ASRs (ASR180, ASR220 and ASR33) and non-functional MotA-ASRs (ASR244, ASR259, ASR266, ASR332, ASR440 and ASRN65), respectively. Both the functional and non-

functional MptA-ASRs were co-transformed with $MotB_{\Delta 51\text{-}70}$ into a Δ*motAmotB* RP437 strain and grown in LB broth with CAM and AMP antibiotics in a 96-well microtiter plate. 0.2% arabinose was added after three hours of incubation.

```
ECMotA 1 --MLILLGYLVVLGTVFGGYLMTGGSLGALYQPAELVIIAGAGIGSFIVGNNGKAIKGTL 58
ASR180 1 MDMQKIIGIVIIFGCVFGGYLMAGGKLDVIWQPAELMIIGGAGVGAFIIGNPLTVIKETA 60
ASR220 1 MDMAKIIGIIVVFASVLGGYVLSHGKIAALIQPFEVLIIGGAAFGAFLQANPGHMTMHVI 60
ASR333 1 --MFAIIGIIVVFACVFGGFLVAGGHLGVLWQPFELLIIGGAALGAFIISNPAKVLKATG 58
ASRN41 1 --MLVIIGIIVVLGSVLGGYVLSHGKLAALIQPAELLIIGGAAIGAFLVANPGKVIKATV 58
           *  ::* ::::. *:**:::: * : .: ** *::**.**..*:*: .*        .
ECMotA 59 KALPLLFRRSKYTKAMYMDLLALLYRLMAKSRQMGMFSLERDIENPRESEIFASYPRILA 118
ASR180 61 NGLGKVFKGPKWKEEHYRDLLALLYELMKTVRSKGLIALEEHIENPQESSIFNRYPKVLK 120
ASR220 61 KKSMKMFGGSRFSHAYYLEVLGLVYEILNKSRREGMMAIEADIEDPAASPIFAKYPTVLK 120
ASR333 59 KALAKVFKGSKYKKEDYLELLALLYELFQTARKEGPMALEKHIEDPHESPIFQQYPKFLK 118
ASRN41 59 KGLMKVFRGSKYSKADYLDLLALLYELLNKSRREGMMAIEADIENPAESPIFSKYPKILA 118
           :    :*   ::..  * ::*.*:*.:: . *  * :::* .**:*  * **  ** .*
ECMotA 119 DSVMLDFIVDYLRLIISGHMNTFEIEALMDEEIETHESEAEVPANSLALVGDSLPAFGIV 178
ASR180 121 DHQLVSFICDNLRLMVMGNMDPHEIEGIMEQEIEAIEEDLLKPSHALQSMGDALPAFGIV 180
ASR220 121 DERMTAFICDYLRIMSSGNMAPHELEGLFDMELLSMKEELEHPSHAVTGIADGMPGFGIV 180
ASR333 119 DHHAVHFLCDTLRLIVSGSMNPHEVEDLMDEEIETHHHEQHQPAHAIQTVADGLPALGIV 178
ASRN41 119 DHHLVDFICDYLRMMVSGNMDPHEIEGLMDMEIETMHHEAEVPAHALTKVADGLPGFGIV 178
            *     *: * **::  * *  .*:* ::: *: : . :   *::::  :.*.:*.:***
ECMotA 179 AAVMGVVHALGSADRPAAELGALIAHAMVGTFLGILLAYGFISPLATVLRQKSAETSKMM 238
ASR180 181 AAVLGIIKTMGSIDESPAVIGAKIAAALVGTFLGVFMAYGLLGPLATRLEAQVEKEGALY 240
ASR220 181 AAVLGIVVTMASLGGDQAAIGMHVGAALVGTFFGILAAYGFFGPLATSLEHDAKEELNLY 240
ASR333 179 AAVLGVVKTMGSLNEPPEKLGHLIASALVGTFLGVFLAYGFVGPLATKLKQKVDEEAKYF 238
ASRN41 179 AAVLGVVITMGSLGGPQEEIGHHVGAALVGTFLGILLAYGFVGPLATSLEHRAEEETKMY 238
            ***:*:: ::.* .      :*  :. *:****:*:: ***:..**** *.     :
ECMotA 239 QCVKVTLLSNLNGYAPPIAVEFGRKTLYSSERPSFIELEEHVRAVKNPQQQTTTEEA- 295
ASR180 241 KIVKAVLVAHLHGNAPQIAVEAGRKTIPSDHRPSFAELEEALTEQPGEGAGKAAPKAA 298
ASR220 241 EAIKASLVASASGMPPSLAVEFGRKVLYPKHRPSFAELEQAVRGRKSAAPGAAGSEAA 298
ASR333 239 HCIKAALLALQHGYPPQVCVEYARKALYPEERPSFE---------------------- 274
ASRN41 239 QAIKVALVASVNGYPPQLAVEFGRKALPSNVRPSFAELEEAVRGRKAPASQATEEEAE 296
            . :*. *::   *  * :.** .**.:  . ****
```

Supplementary Figure 9: Multiple sequence alignment of WT *E. coli* MotA and the four functional MotA-ASRs (ASR180, ASR220, ASR333 and ASRN41). Below each site (i.e., position) of the protein sequence alignment is a key denoting conserved sites (*), sites with conservative replacements (:), sites with semi-conservative replacements (.), and sites with non-conservative replacements ( ).

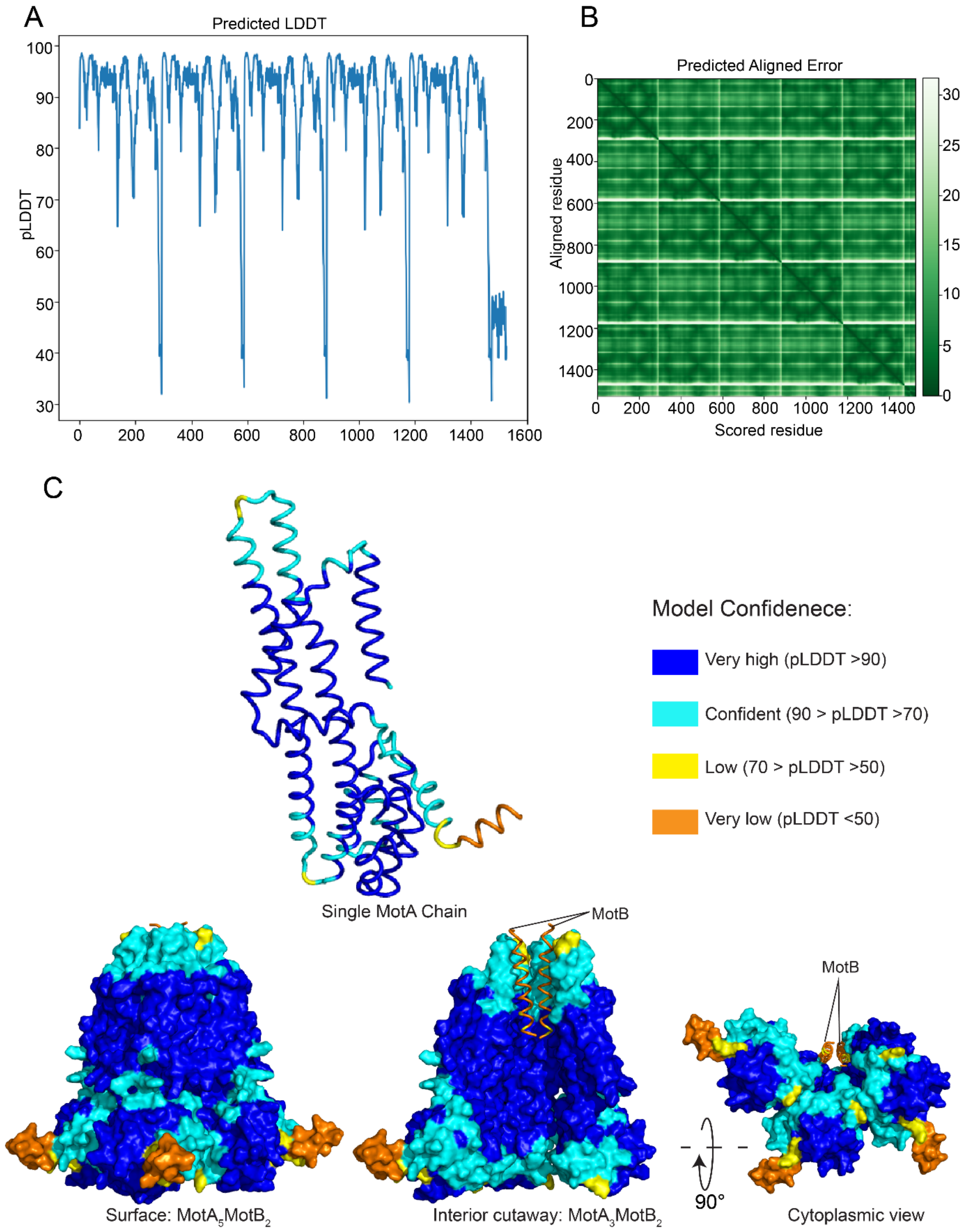

Supplementary Figure 10: Alphafold structure prediction of WT *E. coli* MotAB complex (5:2). (A) Predicted LDDT per position for five copies of MotA (295 residues) and two copies of MotB (26 residues, TM domain). (B) Predicted aligned error (PAE) for the $MotA_5MotB_2$ model. (C) *E. coli* MotAB Alphafold model colour-coded by the pLDDT confidence scores.

The confidence of the model is shown on a single MotA chain in the cartoon representation (upper left) as well as on the surface (lower left) and the interior of the MotAB complex (lower middle and lower right).

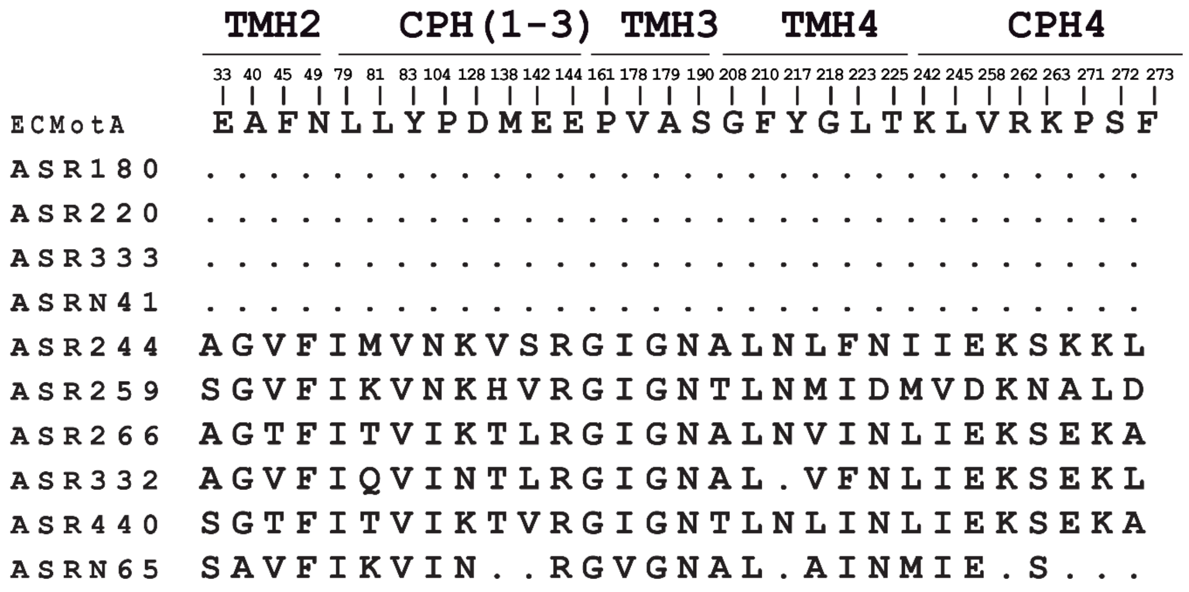

Supplementary Figure 11: 30 proposed critical residues identified from sequence comparison between WT *E. coli* MotA, and both functional and non-functional MotA-ASRs. Sites were identified by those sites which were conserved across functional MotA-ASRs but different in non-functional MotA-ASRs. Location on the MotA regions are are labelled on top: transmembrane helix 2 (TMH2), cytoplasmic helix 1-3 (CPH1-3), transmembrane helix 3 (TMH3), transmembrane helix 4 (TMH4) and cytoplasmic helix 4 (CPH4). Identical residues are presented with dot (.).

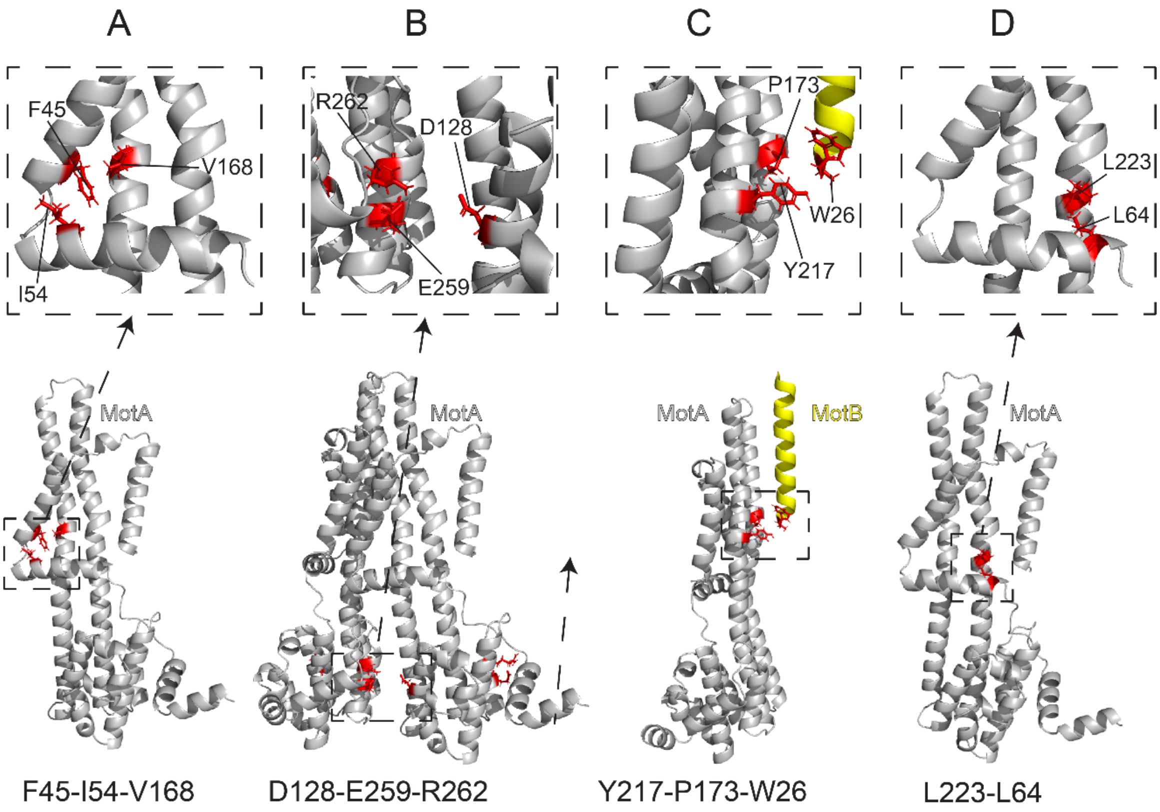

Supplementary Figure 12: Predicted molecular contacts of critical residues. Grey color represents MotA and Yellow color represents MotB. (A) Interaction of MotA-F45 with MotA-I54 and MotA-V168. (B) Interaction of MotA-D128 with other MotA-E259 and R262. (C) Interaction of MotA-Y217 with MotA-P173 and MotB-W26. (D) Interaction of MotA-L223 with MotA-L64.

Supplementary Table 1:

RMSD for each chain of *E. coli* MotAB Alphafold model in comparison with each of 6YKP, 6YKM and 6YSL.

E. coli MotAB	6YKP	RMSD
Chain A (MotA1)	Chain A (MotA1)	2.157
Chain B (MotA2)	Chain B (MotA2)	2.14
Chain C (MotA3)	Chain C (MotA3)	2.263
Chain D (MotA4)	Chain D (MotA4)	2.008
Chain E (MotA5)	Chain E (MotA5)	2.337
Chain F (MotB1)	Chain F (MotB1)	3.517
Chain G (MotB2)	Chain G (MotB2)	3.51
E. coli MotAB	6YKM	RMSD
Chain A (MotA1)	Chain A (MotA1)	2.251
Chain B (MotA2)	Chain B (MotA2)	2.19
Chain C (MotA3)	Chain C (MotA3)	2.299
Chain D (MotA4)	Chain D (MotA4)	2.068
Chain E (MotA5)	Chain E (MotA5)	2.306
Chain F (MotB1)	Chain F (MotB1)	3.516
Chain G (MotB2)	Chain G (MotB2)	3.424
E. coli MotAB	6YSL	RMSD
Chain A (MotA1)	Chain C (MotA1)	1.926
Chain B (MotA2)	Chain D (MotA2)	2.651
Chain C (MotA3)	Chain E (MotA3)	2.077
Chain D (MotA4)	Chain F (MotA4)	2.745
Chain E (MotA5)	Chain G (MotA5)	2.233
Chain F (MotB1)	Chain A (MotB1)	3.386
Chain G (MotB2)	Chain B (MotB2)	3.178

Supplementary Table 2:

List of molecular contacts of the identified 30 conserved MotA residues determined in the alphafold structure of *E. coli* MotA/MotB stator complex measured using pymol (V4.6.0). Inter-subunit contacts are shown in bold.

Conserved residue	Interacting residue	Estimated distance between the residues showed in angstrom (Å)
MotA-E33	MotA-G208	2.9
MotA-E33	MotA-A179	2.4
**MotA-A40**	**MotA-V14**	2.5
MotA-F45	MotA-I54	2.8
MotA-F45	MotA-V168	2.5
MotA-N49	MotA-L167	4.3
MotA-L79	MotA-V258	2.8
MotA-L81	MotA-I109	2.3
MotA-Y83	MotA-P255	2.8
MotA-P104	MotA-V127	3
**MotA-D128**	**MotA-E259**	7.1
**MotA-D128**	**MotA-E262**	4.9
**MotA-M138**	**MotA-Y252**	3.3
MotA-E142	MotA-L246	2.8
MotA-E144	MotA-V243	5.8
MotA-P161	MotA-A60	3
**MotA-V178**	**MotA-F210**	2.4
MotA-A179	MotA-G208	2.5
MotA-S190	MotA-L198	3.2
MotA-Y217	MotA-P173	3

MotA-Y217	MotB-W26	4.1
MotA-G218	MotA-V10	2.4
MotA-L223	MotA-L64	2.7
MotA-L223	MotA-L165	3.7
**MotA-T225**	**MotA-G48**	3.3
MotA-K242	MotA-D148	2.6
MotA-L245	MotA-Y129	3.2
MotA-L245	MotA-M147	2.3
**MotA-K263**	**MotA-Y129**	3.9
MotA-P271	MotA-R262	2.8
MotA-F273	MotA-E259	2.5

Supplementary Table 3:

List of the point mutants and their motility status that we generated in this study. 6PMA = G40A + G40A + I178V + G179A + N217Y + L218G + E262R

**Point mutant name**	**Motility status**	**Swimming ring production**
E. coli MotA-V178I	Motile	+
E. coli MotA-A179G	Motile	+
E. coli MotA-Y217N	Non-motile	-
ASR180-V178I	Motile	+
ASR180-A179G	Motile	+
ASR180-Y217N	Non-motile	-
ASR220-V178I	Motile	+
ASR220-A179G	Motile	+
ASR220-Y217N	Non-motile	-
ASR333-V178I	Motile	+
ASR333-A179G	Motile	+
ASR333-Y217N	Non-motile	-
ASRN41-V178I	Motile	+
ASRN41-A179G	Motile	+
ASRN41-Y217N	Non-motile	-
ASR244-G40A	Non-motile	-
ASR244-I178V	Non-motile	-
ASR244-G179A	Non-motile	-
ASR244-N217Y	Non-motile	-
ASR244-L218G	Non-motile	-
ASR244-E262R	Non-motile	-

ASR244-6PMA	Non-motile	-
ASR259-G40A	Non-motile	-
ASR259-I178V	Non-motile	-
ASR259-G179A	Non-motile	-
ASR259-N217Y	Non-motile	-
ASR259-M218G	Non-motile	-
ASR259-E262R	Non-motile	-
ASR259-6PMA	Non-motile	-
ASR266-G40A	Non-motile	-
ASR266-I178V	Non-motile	-
ASR266-G179A	Non-motile	-
ASR266-N217Y	Non-motile	-
ASR266-V218G	Non-motile	-
ASR266-E262R	Non-motile	-
ASR266-6PMA	Non-motile	-
ASR332-G40A	Non-motile	-
ASR332-I178V	Non-motile	-
ASR332-G179A	Non-motile	-
ASR332-N217Y	Non-motile	-
ASR332-V218G	Non-motile	-
ASR332-E262R	Non-motile	-
ASR332-6PMA	Non-motile	-
ASR440-G40A	Non-motile	-
ASR440-I178V	Non-motile	-
ASR440-G179A	Non-motile	-

ASR440-N217Y	Non-motile	-
ASR440-L218G	Non-motile	-
ASR440-E262R	Non-motile	-
ASR440-6PMA	Non-motile	-
ASRN65-G40A	Non-motile	-
ASRN65-I178V	Non-motile	-
ASRN65-G179A	Non-motile	-
ASRN65-N217Y	Non-motile	-
ASRN65-A218G	Non-motile	-
ASRM65-E262R	Non-motile	-
ASRM65-6PMA	Non-motile	-

Supplementary Table 4:

List of strains and plasmids

**Strain**	**Description**	**Reference**
RP437-ΔMotA/MotB	*E. coli* (ΔMotA, ΔMotB)	This study
RP3087-ΔMotA	*E. coli* (ΔMotA)	
**Plasmids**	**Description**	**Reference**
pSHU1234	PomA and PotB, Ara, CAM^R	Kojima et al., 2008
pBAD33	Empty vector, CAM^R	Guzman et al., 1995
pDB108	MotA and MotB, CAM^R	David F Blair
pMotB	pDB108 ΔMotA CAM^R	Islam et al., 2020
pMotB	pDFB27 ΔMotA AMP^R	David F Blair
pNT8	pSBETa- motB$_1^{Aa}$, KAN^R	Takekawa et al., 2015
pNT9	pSBETa- motB$_2^{Aa}$, KAN^R	Takekawa et al., 2015
P180	PotB and MotA-ASR180, pSHU1234 backbone, CAM^R	This study
P220	PotB and MotA-ASR220, pSHU1234 backbone, CAM^R	This study

P244	PotB and MotA-ASR244, pSHU1234 backbone, CAM^R	This study
P259	PotB and MotA-ASR259, pSHU1234 backbone, CAM^R	This study
P266	PotB and MotA-ASR266, pSHU1234 backbone, CAM^R	This study
P332	PotB and MotA-ASR332, pSHU1234 backbone, CAM^R	This study
P333	PotB and MotA-ASR333, pSHU1234 backbone, CAM^R	This study
P440	PotB and MotA-ASR440, pSHU1234 backbone, CAM^R	This study
pN41	PotB and MotA-ASRN41, pSHU1234 backbone, CAM^R	This study
pN65	PotB and MotA-ASRN65, pSHU1234 backbone, CAM^R	This study
p758	MotB-ASR758, pSHU1234 backbone, CAM^R	Islam et al., 2020
p759	MotB-ASR759, pSHU1234 backbone, CAM^R	Islam et al., 2020
p760	MotB-ASR760, pSHU1234 backbone, CAM^R	Islam et al., 2020
p765	MotB-ASR765, pSHU1234 backbone, CAM^R	Islam et al., 2020
p908	MotB-ASR908, pSHU1234 backbone, CAM^R	Islam et al., 2020
p981	MotB-ASR981, pSHU1234 backbone, CAM^R	Islam et al., 2020
p1024	MotB-ASR1024, pSHU1234 backbone, CAM^R	Islam et al., 2020
P1170	MotB-ASR1170, pSHU1234 backbone, CAM^R	Islam et al., 2020
p1239	MotB-ASR1024, pSHU1234 backbone, CAM^R	Islam et al., 2020
p1246	MotB-ASR1246, pSHU1234 backbone, CAM^R	Islam et al., 2020
p1457	MotB-ASR1457, pSHU1234 backbone, CAM^R	Islam et al., 2020
p1459	MotB-ASR1459, pSHU1234 backbone, CAM^R	Islam et al., 2020
p1501	MotB-ASR1501, pSHU1234 backbone, CAM^R	Islam et al., 2020

Supplementary Table 5:

List of primer sequences and PCR conditions used in this work.

Primer name	Primer sequence
180 GBLOCK - FW	GGAGTGCTTTATGGATATGCAGAAAATTATTGGTATC
180 PSHU - RV	CTGCATATCCATAAAGCACTCCTCACGC
180 PSHU - FW	GAAAGCAGCATAACTTGGAGAATTCATATGGATGAT
180 GBLOCK - RV	ATTCTCCAAGTTATGCTGCTTTCGGGG
220 GBLOCK - FW	AGTGCTTTATGGACATGGCTAAAATCATCG
220 PSHU - RV	ATGTCCATAAAGCACTCCTCACGC
220 PSHU - FW	AGCAGCCTAACTTGGAGAATTCATATGGATGAT
220 GBLOCK - RV	CAAGTTAGGCTGCTTCGCTACC
224 GBLOCK - FW	AGGAGTGCTTTGAATCTATGCTTATTTTGTTGGGATAC
224 PSHU - RV	TAAGCATAGATTCAAAGCACTCCTCACGC
224 PSHU - FW	GAGGCAGCATAACTTGGAGAATTCATATGGATGATG
224 GBLOCK - RV	CTCCAAGTTATGCTGCCTCCTCC
259 GBLOCK - FW	GAGGAGTGCTTTATGGATTTGGCGACGC
259 PSHU - RV	CCAAATCCATAAAGCACTCCTCACGC
259 PSHU - FW	AGAAGGAGCATAACTTGGAGAATTCATATGGATGAT
259 GBLOCK - RV	TTCTCCAAGTTATGCTCCTTCTGCTGC
244 GBLOCK - FW	GGAGTGCTTTATGGACATGGCGACAATC
244 PSHU - RV	CCATGTCCATAAAGCACTCCTCACGC
244 PSHU - FW	AGAAGGAGCGTAACTTGGAGAATTCATATGGATGA
244 GBLOCK - RV	TCCAAGTTACGCTCCTTCTGCGG
N41 GBLOCK - FW	GGAGTGCTTTATGCTTGTGATCATCGGG
N41 PSHU - RV	TCACAAGCATAAAGCACTCCTCACGC

N41 PSHU - FW	GGAAGAAGCATAACTTGGAGAATTCATATGGATGAT
N41 GBLOCK - RV	ATTCTCCAAGTTATGCTTCTTCCTCAGTGG
N65 GBLOCK - FW	AGGAGTGCTTTATGGATTTATCAACGATCATCGG
N65 PSHU - RV	GTTGATAAATCCATAAAGCACTCCTCACGC
N65 PSHU - FW	CTTGCGTGGATAACTTGGAGAATTCATATGGATGATG
N65 GBLOCK - RV	TCCAAGTTATCCACGCAAGGCC
266 GBLOCK - FW	GGAGTGCTTTATCGCGACTATCATCGG
266 PSHU - RV	TAGTCGCGATAAAGCACTCCTCACGC
266 PSHU - FW	GCCGAGTGACTTGGAGAATTCATATGGATGATG
266 GBLOCK - RV	TTCTCCAAGTCACTCGGCTTTCTCG
332 GBLOCK - FW	AGGAGTGCTTTATTACAACGATTATTGGCTTAGTTTTAGG
332 PSHU - RV	CAATAATCGTTGTAATAAAGCACTCCTCACGC
332 PSHU - FW	AGCTGGAATGACTTGGAGAATTCATATGGATGATG
332 GBLOCK - RV	ATTCTCCAAGTCATTCCAGCTTTTCGCG
333 GBLOCK - FW	AGGAGTGCTTTATGTTTGCAATTATCGGGATCAT
333 PSHU - RV	GATAATTGCAAACATAAAGCACTCCTCACGC
333 PSHU - FW	CGTTCGAATGACTTGGAGAATTCATATGGATGATG
333 GBLOCK - RV	AATTCTCCAAGTCATTCGAACGAGGGAC
440 GBLOCK - FW	GGAGTGCTTTATCGCCACTATTATCGGATTG
440 PSHU - RV	TAGTGGCGATAAAGCACTCCTCACGC
440 PSHU - FW	AAGCAGAGTGACTTGGAGAATTCATATGGATGATGAA
440 GBLOCK - RV	GAATTCTCCAAGTCACTCTGCTTTCTCGC
WT-VA178-79IG-F	CCCATTACAGCCCCAATAATACCAAACGCCGGAAGTG
WT-VA178-79IG-R	CACTTCCGGCGTTTGGTATTATTGGGGCTGTAATGGG
WT-I79G-F	CCCATTACAGCCCCAATAATACCAAACGCCGGAAGTG

WT-I79G-R	CACTTCCGGCGTTTGGTATTATTGGGGCTGTAATGGG
WT-Y217N-F	GGAAATAAATCCGTTAGCCAATAAAATGCCGAGGAAAGTC
WT-Y217N-R	GACTTTCCTCGGCATTTTATTGGCTAACGGATTTATTTCC
180-VA178-79IG-F	AGGACTGCGCCAATAATGCCGAACGCGGGCA
180-VA178-79IG-R	TGCCCGCGTTCGGCATTATTGGCGCAGTCCT
180-Y217N-F	CAAGTAACCCGTTAGCCATGAAGACTCCCAGAAATG
180-Y217N-R	CATTTCTGGGAGTCTTCATGGCTAACGGGTTACTTG
220-VA178-79IG-F	CCCAAAACGGCACCTATAATTCCAAAGCCAGGCATACCAT
220-VA178-79IG-R	ATGGTATGCCTGGCTTTGGAATTATAGGTGCCGTTTTGGG
220-Y217N-F	GGACCAAAGAAGCCGTTCGCTGCCAAAATACCG
220-Y217N-R	CGGTATTTTGGCAGCGAACGGCTTCTTTGGTCC
333-VA178-79IG-F	CCCAATACCGCTCCTATAATCCCAAGAGCGGGTAAGCC
333-VA178-79IG-R	GGCTTACCCGCTCTTGGGATTATAGGAGCGGTATTGGG
333-Y217N-F	GGCCAACAAAGCCGTTGGCTAAGAACACGCC
333-Y217N-R	GGCGTGTTCTTAGCCAACGGCTTTGTTGGCC
N41-VA178-79IG-F	CCCTAAAACGGCACCTATAATACCGAATCCGGGCAATCCG
N41-VA178-79IG-R	CGGATTGCCCGGATTCGGTATTATAGGTGCCGTTTTAGGG
N41-Y217N-F	AGGCCCAACAAATCCATTAGCAAGAAGGATTCCCA
NL 244 YG F	TGGCGAACGGAAGGAAAAGACCATAAGCGGAAAAGATGCCATAAAGGGTG
NL 244 YG R	CACCCTTTATGGCATCTTTTCCGCTTATGGTCTTTTCCTTCCGTTCGCCA
IG 244 VA F	GATCACCGCAGCAACAATACCCATGGTGGGGGC
IG 244 VA R	GCCCCCACCATGGGTATTGTTGCTGCGGTGATC
NM 259 YG F	AGCAATAGGAAAGAATACCCCATAACTCAGAATTGCACCATATAAGGTCGTAAGCAATGC

NM 259 YG R	GCATTGCTTACGACCTTATATGGTGCAATTCTGAGTTATGGGGTATTCTTTCCTATTGCT
IG 259 VA F	CCAAACCTACTAATGTTGCAACCATCCCCATGGCCGGT
IG 259 VA R	ACCGGCCATGGGGATGGTTGCAACATTAGTAGGTTTGG
NV 266 YG F	CGATGGGCAAAAAGATGCCATACGCACTTGCGACACCAT
NV 266 YG R	ATGGTGTCGCAAGTGCGTATGGCATCTTTTTGCCCATCG
IG 266 VA F	CGCGCCTACGATGGGTATTGTCGCAACTGTGATGGG
IG 266 VA R	CCCATCACAGTTGCGACAATACCCATCGTAGGCGCG
V 332 G F	CGAAGGGGCCAAATAAACCATACGCGGAGAAGATC
V 332 G R	GATCTTCTCCGCGTATGGTTTATTTGGCCCCTTCG
IG 332 VA F	GCCCCATTACAGCCGCAACGATCCCCATAGTCG
IG 332 VA R	CGACTATGGGGATCGTTGCGGCTGTAATGGGGC
NL 440 YG F	TGTTCGCGATGGGCAAAAAGATCCCATACGCAAGAATTGCCCCATACAATG
NL 440 YG R	CATTGTATGGGGCAATTCTTGCGTATGGGATCTTTTTGCCCATCGCGAACA
IG 440 VA F	CGCCCGCATTCGGTATGGTTGCAACACTTATTGGTCTTG
IG 440 VA R	CAAGACCAATAAGTGTTGCAACCATACCGAATGCGGGCG
244-G40A-F	AACCGCTCCGATTGTAGCGCCGAAAACGATAAG
244-G40A-R	CTTATCGTTTTCGGCGCTACAATCGGAGCGGTT
244-F49N-F	CTTAATTTCATTCATTGGGTTAGAAATCAGAACCGCTCCGATTGTAC
244-F49N-R	GTACAATCGGAGCGGTTCTGATTTCTAACCCAATGAATGAAATTAAG
244-K262S,S263K-F	CTCATTGGGCGGAAGATACTTTCTCAATTTCTCCTCAATAACACGCG

244-K262S,S263K-F	CGCGTGTTATTGAGGAGAAATTGAGAAAGTATCTTCCGCCCAATGAG
259-G40A-F	TAAGGACAACAAAGATAGAAGCTCCCACTACGATAAGAATG
259-G40A-R	CATTCTTATCGTAGTGGGAGCTTCTATCTTTGTTGTCCTTA
259-F49N-F	GCTCCAAAAAATTGTCCCATAGTGTTCTTCATAAGGACAACAAAGATAGA
259-F49N-R	TCTATCTTTGTTGTCCTTATGAAGAACACTATGGGACAATTTTTTGGAGC
259-K262S,S263K-F	CGCGTTTACTCTCGTTTAAATACTTCCTCAAATAACTGTCAATCACGCG
259-K262S,S263K-F	CGCGTGATTGACAGTTATTTGAGGAAGTATTTAAACGAGAGTAAACGCG
266-G40A-F	TCGTGGCACCAATCGTGGCTCCAAATACAATGATG
266-G40A-R	CATCATTGTATTTGGAGCCACGATTGGTGCCACGA
266-F49N-F	TTCAGTTCTTTCAGAGGGTTCGATACCATCGTGGCACC
266-F49N-R	GGTGCCACGATGGTATCGAACCCTCTGAAAGAACTGAA
266-K262S,S263K-F	GCGCTCCTTTGGTGGTAAAAACTTTCTTAATTTTTCTTCGATGATGCGTGGATTTTCACC
266-K262S,S263K-F	GGTGAAAATCCACGCATCATCGAAGAAAAATTAAGAAAGTTTTTACCACCAAAGGAGCGC
332-G40A-F	TCACAGCACCGATAGTCGCGCCAAAAATGATAATG
332-G40A-R	CATTATCATTTTTGGCGCGACTATCGGTGCTGTGA
332-F49N-F	TTCCTTCATGGGATTTGACACCATCACAGCACCGATAGTC
332-F49N-R	GACTATCGGTGCTGTGATGGTGTCAAATCCCATGAAGGAA
332-K262S,S263K-F	TTCTTTGGGGCTTAAGTACTTCCTTAACTTTTCTTCGATTACACGCGGGTTTTCGCC
332-K262S,S263K-F	GGCGAAAACCCGCGTGTAATCGAAGAAAAGTTAAGGAAGTACTTAAGCCCCAAAGAA

266-K262S,S263K-F	GCGCTCCTTTGGTGGTAAAAACTTTCTTAATTTTTCTTCGATGATGCGTGGATTTTCACC
266-K262S,S263K-F	GGTGAAAATCCACGCATCATCGAAGAAAAATTAAGAAAGTTTTTACCACCAAAGGAGCGC
332-G40A-F	TCACAGCACCGATAGTCGCGCCAAAAATGATAATG
332-G40A-R	CATTATCATTTTTGGCGCGACTATCGGTGCTGTGA
332-F49N-F	TTCCTTCATGGGATTTGACACCATCACAGCACCGATAGTC
332-F49N-R	GACTATCGGTGCTGTGATGGTGTCAAATCCCATGAAGGAA
332-K262S,S263K-F	TTCTTTGGGGCTTAAGTACTTCCTTAACTTTTCTTCGATTACACGCGGGTTTTCGCC
332-K262S,S263K-F	GGCGAAAACCCGCGTGTAATCGAAGAAAAGTTAAGGAAGTACTTAAGCCCCAAAGAA
Plug Del Fs	CTAATAGTGAAAGCCCAATTCCCGG
Plug Del F-T	ACGGGCGGCGATCGCATTTCTAATAGTGAAAGCCCAATTCCCGG
Plug Del Rs	GGAGATGGAGATCAGCCACATCA
Plug Del R-T	AAATGCGATCGCCGCCCGTGGAGATGGAGATCAGCCACATCA

PCR conditions for linear vector preparation:

Step	Temperature	Time
Initial Denaturation	98°C	2 mins
X 25 Cycles	98°C<br>65°C<br>72°C	30 Sec<br>1 min<br>3 mins 30 Sec
	72°C	5 mins

PCR conditions for MotA insert preparation:

Step	Temperature	Time
Initial Denaturation	98°C	2 mins
X 15 Cycles	98°C<br>65°C<br>72°C	30 Sec<br>1 min<br>3 mins 30 Sec
	72°C	5 mins
